# Are Ancestral Medical Practices the Future Solution to Today’s Medical Problems?

**DOI:** 10.3390/molecules26154701

**Published:** 2021-08-03

**Authors:** José A. Lupiáñez, Eva E. Rufino-Palomares, Amalia Pérez-Jiménez

**Affiliations:** 1 Department of Biochemistry and Molecular Biology I, Faculty of Sciences, University of Granada, Avenida Fuentenueva, 118071 Granada, Spain; 2 Department of Zoology, Physiology and Ecology, Faculty of Sciences, University of Granada, Avenida Fuentenueva, 118071 Granada, Spain

Our cells and organs are threatened and, in most cases, constantly subjected to the aggression of numerous situations, both endogenous, characterized by unfavorable genetics, and exogenous, by deficient or inadequate nutrition, and even by a hostile environment; in most cases, they ultimately cause a cascade of degenerative and cardiovascular diseases, cancer, and infections, as well as those related to the metabolic syndrome, all of which eventually generate irreversible damage to the organism and, consequently, a significant deterioration in its survival. In many of these cases, exogenous treatment with essential biocomponents present in numerous species, mainly plants, can reverse this deterioration and, therefore, increase survival.

The year 2020 marked a millennium since the publication of the two books that laid the foundations of modern and current medicine—the volumes entitled, The Book of Healing and The Canon of Medicine, the latter commonly known as the “Avicenna Codex.” Both books were published between 1014 and 1020 by the Persian Muslim physician, polymath, philosopher, scientist, and astronomer Abū ‘Alī al-Husayn ibn’ Abd Allāh ibn Sĩnã, better known as Ibn Sina and westernized as Avicenna, as well as the “Prince of the Sages,” the greatest of physicians, the Master par excellence, or the Third Master (after Aristotle and Al-Farabi). Although it is evident that Avicenna put into practice the first principles of surgery, his greatest contribution was the use of the main natural chemical components, derived from the plant and animal (kingdoms?) world, to achieve the cure of the most important diseases of the time as described in the second book of the Avicenna Codex, entitled De Medicinis Simplicibus: Pharmacologicae de Herbis Medicinalibus, which deals with the pharmacology of medicinal herbs and is intended for the study and use of natural medicines [1]. However, we must recognize that numerous types of plasters formed from plant extracts have been mainly used by humankind practically from the Metal Age (6000 BC–3300 BC) until almost the end of the Modern Age (1492 AD–1789 AD) and have been especially important in the development of the different traditional medicines of different cultures, both for Western and, especially, for Oriental medicines.

At present, and especially since the last 20 years, there is a real explosion in the search for natural chemical compounds, generally known as “phytochemicals,” from the plant (kingdom?) world, both terrestrial and aquatic, capable of presenting important and abundant biological properties. The so-called phytochemicals are chemical compounds produced by many botanical species and which play an important role in the growth of the plant species themselves and also as participants in the defense against competitors, pathogens, or predators. The word “phytochemical” has been generally used to describe a number of botanical compounds that are being investigated for their effects on health, and many of them are being used both as drugs, in different traditional medicines, and also as poisons [2].

Among the enormous variety of phytochemicals currently characterized, we can find flavonoids, such as anthocyanins, flavones, flavanones, and flavanols (catechins, epicatechins, etc.), phytosterols, terpenoids, lignans, and stilbenes, the last two being considered an excellent source of polyphenols such as resveratrol [3]. All of them have been recognized with a large number of bioactive effects as nutraceuticals, essential nutrients, and even allelopathic, thus influencing the growth, survival, or reproduction of other organisms [4]. Among all these phytochemicals, several should be highlighted, namely, terpenoids and polyphenols, of which consequential research related to their bioactive capacities both in vitro and in vivo is being carried out [5]. Many of these compounds have properties such as anticancer, using different cancer lines, both solid and liquid [6,7,8,9,10,11], antiangiogenic [7,12,13,14,15], antioxidant [16,17,18], anti-inflammatory [19,20], cardio- and neuroprotective [21,22], antidiabetogenic [23,24], antifungal [25], antimicrobial and antiviral [26,27], antiparasitic [28], growth inducing [29,30,31], and enzyme inhibitory or activating [32,33], as well as modulators in the production of reducing equivalents whose role is essential to explain most of the processes of metabolic biosynthesis [34], of cellular and organic growth, nutrition and differentiation processes [35,36,37,38] as well as, of cellular detoxification processes [39] and oxygen-free radical scavenging [18,40].

Currently, special emphasis is put on the search for a great variety of chemical derivatives of all these phytochemicals, finding in many cases a significant increase in the different bioactive capacities with respect to those of the original compound [5]. Numerous chemical groups are being used in the synthesis of chemical derivatives of the most important triterpenoids in order to select derivatives that present a significant increase in efficiency in their bioactivity. Among them, acyl, aminoacyl, and dipeptidyl groups [26,27,41,42], pegylated and pegylated diamine derivatives [43,44,45,46], and even coumarin conjugates [47] stand out. In all cases, the anticancer, anti-inflammatory, antioxidant, or antiviral effects originally present in the molecules from which they are obtained are significantly increased.

This Special Issue entitled Anticancer properties of natural products and derivatives, (https://www.mdpi.com/journal/molecules/special_issues/anticancer_np, accessed on 30 November 2020), has tried to expose part of the works currently carried out in the field of natural products and their bioactivities, mainly anticancer, to the scientific world. It consists of a total of 15 publications, of which 11 are original articles and 4 are bibliographic reviews. All of them cover very different topics, both in terms of the type of active components or nutraceuticals and the organic source that provides them.

Propolis is a resinous mixture collected opportunistically by honeybees from various plant sources, such as tree buds, sap exudates, or other sources, which is then processed within the hive for use as a sealant for small holes to prevent infection. Although it has been used as a traditional and folk medicine for several millennia, numerous biological properties of propolis have recently been described, including cytotoxic, antiviral, antimicrobial, and antioxidant activities that promote the scavenging of oxygen free radicals (ROS) [48]. In this regard, Wezgowiec et al. [49] have studied the chemical composition and anticancer and anti-inflammatory activities, in vitro, of ethanolic and ethanol–hexane extracts of propolis from different Polish regions on tongue cancer cells (SCC-25). High concentrations of polyphenols and flavonoids seem to be responsible for these biological activities and for the differences between the activities of propolis from different locations. Administration of these extracts produced, among other effects, a significant reduction in mitochondrial and proliferative activity, together with a clear modification of oxygen-free radical scavenging activity. All these effects indicate a selective anticancer and anti-inflammatory potential, although, as the authors indicate, further study of the molecular mechanisms that explain this is necessary to obtain promising health benefits.

*Adenosma bracteosum* (Bonati) is a plant belonging to the group of tracheophytes, mainly present in the Southeast Asian region and whose extracts have been used in traditional Vietnamese medicine to cure liver diseases. The main active groups present are polyphenols, terpenoids, and flavonoids. Recently, ethanolic extracts have been shown to have significant antidiabetogenic activity [50]. In this context, Nguyen et al. [51] have analyzed the anticancer capacity of the different fractions derived from the ethanolic extract of this tracheophyte, using two cancer cell lines, one for lung carcinoma (NCI-H460) and the other for liver carcinoma (HepG2). Of all the fractions, the chloroform-derived one seems to be the most active being the active principles that provide this bioactivity are flavonoids, xantomicrol, and its oxygenated derivative, together with the triterpene, ursolic acid. Its most significant activities focus on modulating free radical levels and mitochondrial membrane potential. All these activities, together with potent cytotoxic activity, seem to be responsible for an increase in the levels and therefore the activity of caspase-3, which is ultimately responsible for the increase in cell apoptosis, suggesting that this plant offers a good opportunity to develop new anticancer drugs.

On many occasions, continuous treatment with radioactivity and chemotherapy to different types of cancer, in certain patients, can cause adverse side effects that, on too many occasions, generate great resistance to specific drugs in these tumors; therefore, it is necessary to find more effective and, mainly, less invasive pharmacological treatments. *Moringa oleifera* is a tree native to northern India for which important nutritional and pharmacological functions have been described, such as anti-inflammatory, antihypertensive, diuretic, hepatoprotective, hypocholesterolemic, antispasmodic, antiulcer, and antibacterial [52]. In this context, Luetragoon et al. [53] have investigated the anticancer effects of an active principle, 3-hydroxy-β-ionone, a sesquiterpenoid present in extracts of *Moringa oleifera* leaves. These authors have demonstrated the in vitro anticancer capacity of this compound in epidermoid carcinoma of the head and neck (SCC-15), by detecting cell cycle arrest in the G_2_/M phase and a significant increase in cell apoptosis, thanks to an increase in caspase-3 levels, together with a decrease in the anti-apoptotic protein Bcl-2 and profound inhibition of cell migration after 6 h of treatment.

Among the active compounds present in the fruit and leaf of the olive tree (*Olea europaea* L.), the pentacyclic triterpenes stand out, along with, mainly due to their content, maslinic acid, of which a large number of beneficial health effects have been demonstrated on many occasions [5,8,9,10,16,29,30,31,32]. One of the most controversial functions of maslinic acid is its antioxidant capacity; thus, Mokhtari et al. [18] investigated this property in murine cutaneous melanoma cells (B16F10). In addition to the known selective cytotoxic effects of maslinic acid on cancer cells, it was also demonstrated that after provoking an oxidative stress situation by the addition of hydrogen peroxide (H_2_O_2_), the triterpene isolated from the olive tree protected tumor cells from concomitant oxidative damage by decreasing ROS levels and modifying the activities of different antioxidant enzymes such as superoxide dismutase (SOD), glutathione S-transferase (GST), and glutathione peroxidase (GPX), thus demonstrating a high antioxidant capacity of this triterpene and therefore its beneficial effects on health.

*Elaeagnus angustifolia*, commonly called paradise tree or Bohemian olive, is a shrub native to the Middle East whose floral extracts have been traditionally used to treat many diseases in that area. In their article, Jabeen et al. [54] reveal that flower extracts from the paradise tree were able to inhibit in vitro cell proliferation and modify cell cycle progression in two breast cancer lines (SKBR3 and ZR75-1) positive for the human epidermal growth factor receptor 2 (HER2) protein; furthermore, these extracts inhibited epithelial-mesenchymal transition (EMT), an important event for cancer invasion and metastasis, by increasing the levels of E-cadherin and β-catenin and inhibiting those of vimentin and fascin, as main marker molecules of cell invasiveness. Furthermore, these authors demonstrate the chemopreventive effects of these extracts by blocking HER2 activities and inactivating the JNK/1/2/3 signaling pathway.

As we have stated in this article, natural products play an important role in the development of new nutraceuticals that help to prevent and cure diseases, and in this sense, the triterpenes present in the olive tree (*Olea europaea* L.) are very relevant. An example of these compounds is uvaol, and a practically innovative study of this active principle was carried out by Bonel-Pérez et al. [15], in which they analyzed its anticancer and antimetastatic effects in vitro in a hepatocarcinoma cell line (HepG2). These authors show the main molecular responsible for these activities, focusing their study on the levels of the heat shock protein HSP60, on the levels of ROS, as well as those of the antiapoptotic Bcl2 and proapoptotic Bax proteins, together with a cellular arrest in the G_0_/G_1_ phase and downregulation of the AKT/PI3K and MAPK signaling pathway. These results constitute an interesting challenge in the treatment of this type of cancer.

Another important source of active ingredients, which are increasingly used in conventional oncology, are the so-called medicinal mushrooms, and although they act by interfering with tumor cells, disrupting both the development and progression of the disease, the mechanisms of action that cause this have yet to be elucidated. Roda et al. [55], using triple negative 4T1 mice administered in vivo with a mixture of these medicinal mushrooms, observed a significant reduction in both tumor density and the number of metastatic bodies, showing at the same time a decrease in inflammation and oxidative stress, both in the primary tumor and in metastases. These extraordinary effects molecularly implicate the p53/Bax versus Bcl2/PARP1/PCNA axis, forcing triple-negative breast cancer cells into apoptosis.

*Pogostemon cablin*, commonly known as patchouli, is a plant from which essential oil is extracted from the leaves, rich in sesquiterpenes, which has been used as an antiseptic since ancient times in Asia; in addition, its use as a pharmaceutical product prevents or cures various side effects such as fever, headache, pain, and inflammation. Other studies have revealed different bioactivities, such as antimicrobial and antiviral, anti-inflammatory, anti-aging, and antitumor. Therefore, Huang et al. [56] have investigated the in vitro and in vivo response of human hepatoma cells (HepG2 and MAHLAVU) to treatment with the essential oils of this plant. The in vitro antiproliferative effects are explained by the existence of high cytotoxicity, a cell arrest in G_0_/G_1_, together with an increase of apoptosis, both extrinsic and intrinsic, a decrease of the mitochondrial membrane potential, and a modulation of the Akt/mTOR pathway. In in vivo studies, they used BALB/c nude mice as a xenograft model, demonstrating that the administration of these essential oils suppressed tumor growth, owing to a significant reduction of the VEGF/VEGFR axis and an induction of apoptosis in tumor cells, prolonging the life of the mice.

Lactucoprim is a sesquiterpene lactone, a component of lactucarium, a milky liquid extracted from the wild lettuce, *Lactuca virosa*. It is also used as a sedative and analgesic. Its antiproliferative activity on U87Mg glioblastoma cells in vitro has been analyzed by Rotondo et al. [57]. In addition to a potent cytotoxic effect and cell cycle arrest in G_2_/M, lactucoprim administration showed a significant reduction in cell growth and migration, as well as activation of autophagy. All these findings, together with an increase in apoptosis, owing to a decrease in procaspase-6 levels, an increase in PARP, and its positive participation in oxidative stress, allow us to affirm that lactucoprim can be considered as a promising adjuvant therapy in the treatment of this disease.

In the wild jungles of Costa Rica grow a large number of fungi that contain a large number of molecules with antitumor capacity. One of them, *Macrocybe titans*, contains a triacylglyceride called macrocybin, (which?) whose structure includes oleic acid, palmitic acid, and a more complex fatty acid with two double bonds. Using this active principle, Vilariño et al. [58] studied its anticancer activity in a xenograft with A549 tumors, achieving a significant reduction in tumor growth and a positive regulation of caveolin-1 expression, which explains the disassembly of the actin cytoskeleton in tumor cells.

The following article presents an example of how many of the chemical derivatives of a natural compound could increase both the ability to move specifically to their target and their biological activity. Grymel et al. [59] have synthesized, through the copper-catalyzed 1,3-dipolar azide-alkyne (CuAAC) cycloaddition reaction, new betulin derivatives with monosaccharides via a linker containing a heteroaromatic 1,2,3-triazole ring. These authors tested the in vitro cytotoxicity of all these derivatives using two cancer cell lines, one for human breast carcinoma (MCF-7) and the other for colorectal carcinoma (HCT-116). The main finding of this work is that the idea of adding sugar units to the betulin structure allows an optimized specific transfer of the glycoconjugate to the tumor cells.

Although, for now, we cannot consider phytochemicals and their metabolites as essential nutrients in humans, the fact is that more and more research, and a good example is this Special Issue, strongly links the fact that their intake leads to greater prevention of many diseases, including cancer. In this sense, in their article, Ferraz da Costa et al. [60] review both the molecular mechanisms of grape and red wine bioactive compounds and their metabolites in breast cancer, as well as chemoprevention and its treatment. It is very interesting to note the approach taken, relating the structure of the different compounds, flavonoids, monomeric catechins, proanthocyanidins, anthocyanins, anthocyanidins, and non-flavonoid phenolic compounds, such as resveratrol, to their metabolism and especially to their bioavailability. The review also includes an excellent discussion of in vitro, in vivo, and clinical trials on chemoprevention and therapy with these molecules.

Traditionally, chemotherapy and radiotherapy have been used in the treatment of cancer; however, it is necessary to discover new treatments that, on the one hand, are less aggressive for the organism and, on the other hand, more specific in order to recognize and differentiate tumor cells from those that are not. Possibly one of these novel treatments to eliminate different types of cancer is photodynamic therapy (PDT). The main requirement of this type of therapy is the use of photosensitizers (PS) and photoactivation using a specific wavelength of light in the presence of molecular oxygen. The combined action of these two elements is capable of exerting a cascade of molecular actions that end up modulating processes such as apoptosis, necrosis, and autophagy in tumor cells. Photoactive substances derived from medicinal plants have been shown to be safe in comparison with synthetic compounds, and although more and more natural compounds are being discovered that exhibit photosensitizing potential, it is necessary to continue along this path to find new, more active molecules that cover a broader spectrum. In this regard, Muniyandi et al. [61], in their review, put special emphasis on the importance of common photoactive groups (furanocoumarins, polyacetylenes, thiophenes, curcumin, alkaloids, and anthraquinones), their phototoxic effects, their anticancer activity, and their use as a potent PS to achieve an effective PDT result in the treatment of various types of cancers. Another review related to the use of photosensitizing compounds is presented by Verebová et al. [62]. These authors provide a comprehensive summary of the physical and chemical properties of photosensitizers of the hypericin type and their model composed of emodin, quinizarin, and danthron, and show us important antiviral, antifungal, antineoplastic and antitumor effects. They conclude their work by stating that these compounds can be used as potential agents in photodynamic therapy, especially in cancer therapy.

Following the common pattern of synthesizing chemical derivatives of natural molecules in the search for more biologically effective compounds, Professor Csuk’s group [63] has screened and reviewed several triterpenoid derivatives of rhodamine. This compound belongs to a group of fluorescents, xanthene-based, fluorescein-derived, heterocyclic organic molecules that have traditionally been used as dyes and amplifying substances in dye lasers. In their study, these authors reveal the degree of cytotoxicity of these derivatives, all of which exhibit a low nanomolar range, combined with good tumor/non-tumor selectivity. The studies indicate that the homopiperazinyl spacer is more effective than the piperazinyl spacer, which allows them to state that the use of a homopiperazinyl spacer can be considered a promising candidate in biological studies.

As mentioned in this article, more and more new natural compounds with greater efficacy and biological selectivity are being sought, and all of us who form part of the medical–scientific community hope that some can be incorporated into the list of effective drugs that serve to save lives, especially at this time, when a pandemic such as COVID-19 is seriously affecting humanity, although much more severely in those countries with crucial problems in obtaining vaccines, but which, possibly, may have greater possibilities of obtaining these types of drugs. For all the contributions, we are deeply grateful for the effort and collaboration of all the authors who have made it possible, with their articles, to make this Special Issue a reality.

## Data Availability

Not applicable.
